# Ultraviolet phosphorescent carbon nanodots

**DOI:** 10.1038/s41377-022-00837-1

**Published:** 2022-05-20

**Authors:** Shi-Yu Song, Kai-Kai Liu, Qing Cao, Xin Mao, Wen-Bo Zhao, Yong Wang, Ya-Chuan Liang, Jin-Hao Zang, Qing Lou, Lin Dong, Chong-Xin Shan

**Affiliations:** Henan Key Laboratory of Diamond Optoelectronic Material and Devices, Key Laboratory of Material Physics, Ministry of Education, School of Physics and Microelectronics, Zhengzhou University, Zhengzhou, 450001 China

**Keywords:** Optics and photonics, Optical materials and structures

## Abstract

Phosphorescent carbon nanodots (CNDs) have generated enormous interest recently, and the CND phosphorescence is usually located in the visible region, while ultraviolet (UV) phosphorescent CNDs have not been reported thus far. Herein, the UV phosphorescence of CNDs was achieved by decreasing conjugation size and in-situ spatial confinement in a NaCNO crystal. The electron transition from the *p*_x_ to the *sp*^2^ orbit of the N atoms within the CNDs can generate one-unit orbital angular momentum, providing a driving force for the triplet excitons population of the CNDs. The confinement caused by the NaCNO crystal reduces the energy dissipation paths of the generated triplet excitons. By further tailoring the size of the CNDs, the phosphorescence wavelength can be tuned to 348 nm, and the room temperature lifetime of the CNDs can reach 15.8 ms. As a demonstration, the UV phosphorescent CNDs were used for inactivating gram-negative and gram-positive bacteria through the emission of their high-energy photons over a long duration, and the resulting antibacterial efficiency reached over 99.9%. This work provides a rational design strategy for UV phosphorescent CNDs and demonstrates their novel antibacterial applications.

## Abstract (web summary)

Ultraviolet high-energy photons of phosphorescent carbon dots for antibacterial applications. Herein, the UV phosphorescent CNDs were achieved by decreasing conjugation size and spatial confinement. As a demonstration, the CNDs are used to inactivate gram-negative/positive bacteria.

## Graphical abstract

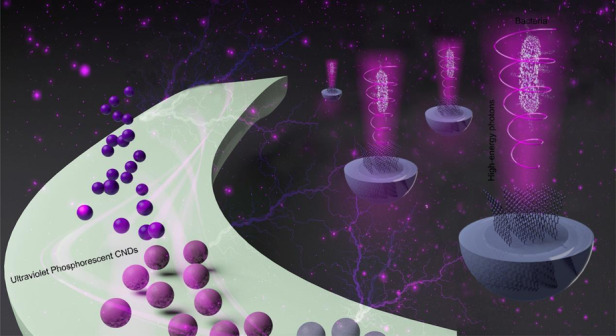

## Introduction

Carbon nanodots (CNDs) as promising phosphorescence candidates develop rapidly^1,2^. Their relatively easy preparation process, unique optical properties, and high biocompatibility make them especially fascinating in the field of bioscience and technology^3–5^. To date, room temperature phosphorescent CNDs have facilitated amazing advances in optoelectronics devices, time-resolved imaging, and phosphorescence bioimaging, due to the efforts of scientists^6–11^. However, most phosphorescence of CNDs is centered in the visible region, and there remains an issue of limited phosphorescence wavelengths. In particular, ultraviolet (UV) phosphorescent CNDs that can emit high-energy photons over a long duration have not been realized. Thus, the development of UV phosphorescent CNDs is of great importance and significance considering their attractive applications in UV light-emitting devices, sterilization and so on^12–14^.

Modulating the wavelength and brightening the triplet excitons of CNDs are the key points for inducing UV phosphorescence. The conjugation size modulation of CNDs as the most accepted method has been used to produce colorful, glowing CNDs. Enlarging the conjugation size of CNDs can lead to longer wavelength emissions due to the decrease in system energy caused by the increase in electron delocalization. The corresponding results have been reported by Qu and Lin et al., and this is significant for near-infrared emissive CNDs^15,16^. Crosslink-enhanced emission (CEE), a novel concept conceived by Yang and coauthors, sheds light on the emission mechanism of CNDs^17^. Cross-linking during the formation of CNDs can also increase their conjugate size to some extent, leading to a narrower bandgap of the synthesized CNDs. Thus, optimizing the carbonization process and decreasing the conjugation size of CNDs will endow them with the desired UV emission properties. As for brightening triplet excitons, “aggregation”, “crystallization” and “confined-domain CEE” concepts were proposed for aggregation-induced emission materials, molecule emission materials, and CND emission materials, the study of which is important for realizing their phosphorescence and understanding the underlying mechanisms^18–20^. The nature of the three concepts is similar, that is, the restriction of the vibration/rotation of the luminophore, which can be described as confinement. The confinement of CNDs within nano spaces has been used for water-soluble phosphorescent CNDs^21^. Thus, UV phosphorescent CNDs can be achieved by combining confinement and conjugation size modulation engineering.

We aim to achieve the UV phosphorescence of CNDs in this work; thus, the formed CNDs should have a small conjugation size and sufficient ISC driving force within a confined environment. According to calculations of the first-principles density functional theory, the bandgaps of configurations consisting of melamine molecules are in the region of 3.7–3.8 eV, indicating that CNDs with a small conjugation size are expected to be formed by using melamine as a precursor. In addition, N atoms with lone pairs of electrons favor the triplet exciton population. By tailoring the size of the CNDs from over 100 to ~3.6 nm, the conjugation size decreases, the phosphorescence emission peak can be tuned to 348 nm, and the lifetime can reach 15.8 ms under the confinement of NaCNO. Due to their high-energy photons and long-lasting time, the CNDs are used to inactivate gram-negative/positive bacteria with antibacterial efficiency exceeding 99.9%, demonstrating their novel antibacterial applications.

## Results

**Fig. 1:**
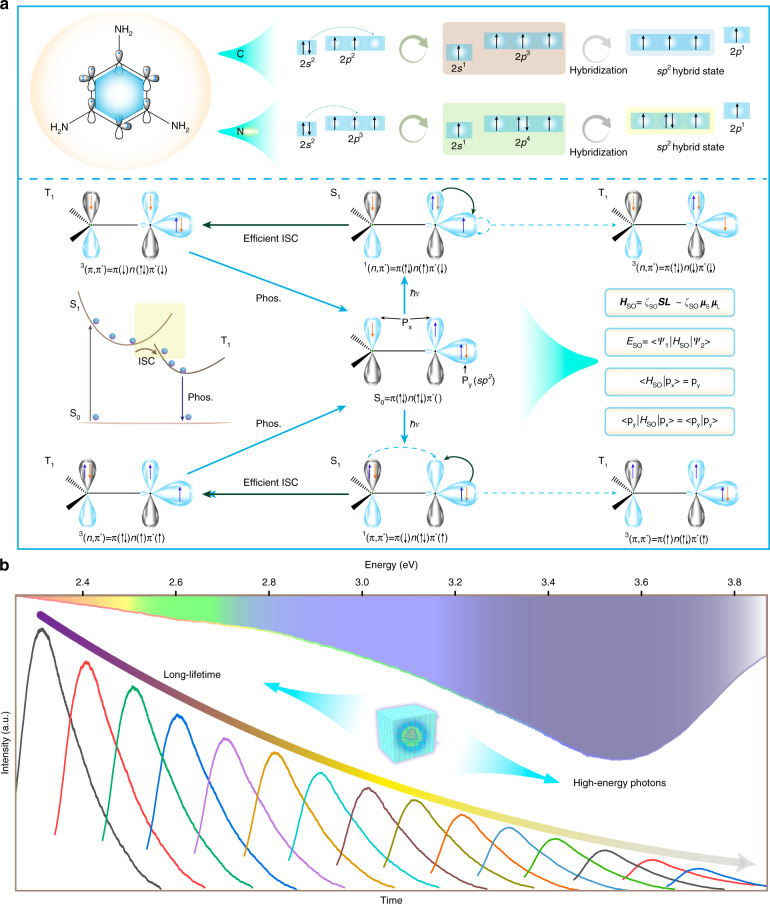
Schematic illustration for achieving UV phosphorescence in CNDs. **a** Molecular structural unit and electron orbitals of the C and N atoms in CNDs. Schematic illustration of the El-Sayed rule and the corresponding phosphorescence decay process. **b** Phosphorescence energy spectra and photoluminescence lifetime decay of the UV phosphorescent CNDs

UV phosphorescent CNDs were prepared by using melamine as the luminophore in terms of electron orbits, which will be discussed carefully below. As indicated in Fig. 1a, the outer-shell electron distributions of the carbon and nitrogen atom orbits are 2*s*^2^2*p*^2^ and 2*s*^2^2*p*^3^, and one *s* orbital and two *p* orbitals form three *sp*^2^ hybrid orbitals in the CNDs^22^. For the nitrogen atoms within the CNDs, the three *sp*^2^ hybrid orbitals are not equivalent, which leads to a lone pair of electrons residing in one of the *sp*^2^ hybrid orbitals^23,24^. This lone pair of electrons is not involved in the π-electron system, thus strengthening the spin-orbit coupling (SOC) because of the inversion asymmetry band structure, and this is beneficial for the realization of phosphorescence^25^. Generally, SOC plays a dominant role in electron spin-flipping in heavy-atom free systems. From the El-Sayed rule, the process of the ISC transition of the^1^(n, π^*^) →^3^(π, π^*^) and ^1^(π, π^*^) → ^3^(n, π^*^) is easy to implement^26^. However, the ISC of the ^1^(n, π^*^) → ^3^(n, π^*^) or ^1^(π, π^*^) → ^3^(π, π^*^) with similar electronic configurations is not effective due to the spin-forbidden transition, which leads to the low efficiency of SOC^27^. Therefore, for the recombination rate of triplet excitons, the ^3^(π, π^*^) triplet has a slow recombination rate compared to ^3^(n, π^*^) due to the spin-forbidden transition^28,29^. In this system, the *n* orbit is one of the *sp*^2^ orbits of the N atom, and the electron transition from the *sp*^2^ to the *p*_x_ orbit or the *p*_x_ to the *sp*^2^ orbit of the N atom will produce a one-unit change in the orbital angular momentum, meeting the required angular momentum of the electron spin-flip (from −1/2 to 1/2; or 1/2 to −1/2)^30,31^, which is necessary for the triplet exciton population of the CNDs. The interaction between the orbit and the spin can be described by the equations in Fig. 1a. $\hat H_{so}$ is the spin-orbit coupling Hamiltonian operator, which is in direct proportion to the product of the magnetic moment of orbit angular momentum (*μ*_*L*_) and spin angular momentum (*μ*_*s*_). The spin-orbit coupling energy (*E*_*so*_) pertains to the overlap of the initial orbit wave functions (*ψ*_1_) and terminative orbit wave functions (*ψ*_2_)^32,33^. A larger *E*_*so*_ indicates stronger spin-orbit coupling. A change in spin angular momentum (*μ*_*S*_) induces orbit distortion, and a change in orbit angular momentum (*μ*_*L*_) can also lead to electron spin-flipping^34,35^. Thus, the distinguishing feature of $\hat H_{so}$ is that it can rotate the corresponding electron wave functions by 90°. In this system, matrix-$\langle p_x{{{\mathrm{|}}}}\hat H_{so}{{{\mathrm{|}}}}sp^2\rangle$, has a large value under the effect of $\hat H_{so}$. Furthermore, the bandgaps of CNDs with different structures were investigated based on first-principles density functional theory calculations. Possible configurations denoted as configuration-1 and configuration-2, are shown in Fig. S1. The bandgaps of the two configurations are 3.769 and 3.801 eV. The high overlap of the highest occupied molecular orbital (HOMO) and the lowest unoccupied molecular orbital (LUMO) leads to the effective recombination of electrons and holes, endowing the CNDs with efficient UV luminescence. To realize UV phosphorescence, the CNDs were placed into a rigid environment provided by the NaCNO crystal to stabilize the produced triplet excitons. The plot of the phosphorescence spectra versus time illustrated in Fig. 1b shows the phosphorescence peaks at ~348 nm, which is the first report of UV phosphorescent CNDs. The lifetime of the CNDs is ~15.8 ms. The total photoluminescence (PL) quantum yield (QY) is about 31.7% and the phosphorescence QY is about 16.2%.

**Fig. 2:**
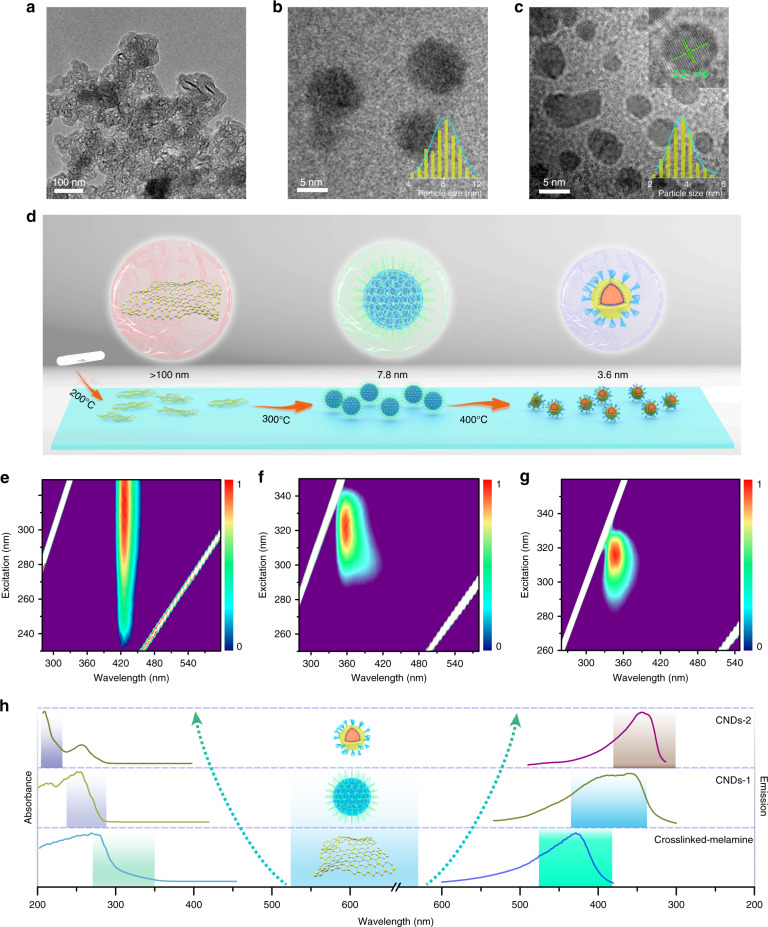
The formation process and fluorescence characterization. TEM images of crosslinked melamine at 200 °C (**a**), CNDs formed at 300 °C (**b**) and CNDs formed at 400 °C (**c**). The insets are the size distributions and high-resolution TEM images of the CNDs. **d** Schematic diagram of the formation mechanism of the CNDs under different conditions. Excitation-fluorescence mapping profiles of crosslinked melamine@Na_2_CO_3_ (**e**), CNDs-1@NaCNO (**f**), and CNDs-2@NaCNO (**g**). **h** The corresponding UV–vis, PL emission spectra of the crosslinked melamine@Na_2_CO_3_, CNDs-1@NaCNO, and CNDs-2@NaCNO

The corresponding morphology and structure evolution of the CNDs were characterized by transmission electron microscopy (TEM), as shown in Fig. 2a–c. Large-scale TEM images indicate that the CNDs have different sizes (Fig. S2), which can clearly reveal the size evolution of the CNDs under different temperatures. Remarkably, the high-resolution TEM image in Fig. 2c shows a clear lattice spacing of 0.21 nm, indicating the formation of CNDs with high crystallinity. Fourier transform infrared (FTIR) spectra and X-ray diffraction (XRD) patterns were collected to further understand the surface and inner structures of the CNDs. A peak at ~27.4° can be observed from the XRD pattern, and the other peaks correspond to Na_2_CO_3_ (owing to the reaction between CO_2_ and NaOH) (Fig. S3a). With increasing temperature, some of the Na_2_CO_3_ may react with the amine groups in the melamine to form NaCNO, the existence of which can be confirmed by the peak centered at 2207 cm^−1^ in the FTIR spectra in Fig. S4. No obvious peaks of the CNDs were observed due to a lower proportion of the CNDs within the NaCNO (Fig. S3b, c). The NaCNO provided a rigid, confining, and isolating environment for the CNDs. An -OH stretching vibrations signals located at 3374 cm^−1^ can be observed, and the peak centered at 3451 cm^−1^ could be assigned to the -NH_2_ surface groups^36,37^. Additionally, the peak centered at ~1800 cm^−1^ originates from the vibration of C=N^38^. Obviously, from the XPS spectra shown in Fig. S5, the peak of N 1 s decreases greatly with increasing synthesis temperature (Table S1), implying that Na_2_CO_3_ can react with amidogen, leading to the collision of the shell and the loss of nitrogen. Thus, a possible formation process of the UV phosphorescent CNDs based on these results is proposed in Fig. 2d. The melamine precursor underwent cross-linking through a polymerization reaction at 200 °C, Na_2_CO_3_ was formed around the crosslinked melamine due to the reaction between CO_2_ and NaOH, and the size of the crosslinked melamine@Na_2_CO_3_ was over 100 nm. When the temperature increased to 300 °C, a highly dense amorphous carbon particle formed, and was accompanied by the formation of NaCNO, resulting in the production of CNDs@NaCNO. The size of the CNDs decreased to ~7.8 nm, and most of the CNDs in this step exhibited an amorphous structure. The high temperature will lead to the aromatization of amorphous carbon particles; when the synthesis temperature increased to 400 °C, the average size of the CNDs decreased to 3.6 nm.

Furthermore, the photophysical properties of the CNDs were investigated. The PL-excitation pattern shows that the luminescence peak at 430 nm changes little with the excitation wavelength, indicating that there is only one kind of luminescence center in the CNDs (Fig. 2e). For the CNDs prepared at 300 °C (denoted as CNDs-1@NaCNO), a luminescence peak was observed at ~360 nm (Fig. 2f). In the case of the CNDs prepared at 400 °C (denoted as CNDs-2@NaCNO), emission peaks were observed at ~342 nm (Fig. 2g). The total PL QYs of the samples synthesized at 200, 300, and 400 °C are 5.3, 12.6, and 31.7%, respectively. Notably, the CNDs showed obvious size-dependent luminescence, and the fluorescence peaks blueshifted from 430 to 342 nm. In detail, the blueshifting of the emission is due to the decreased electron delocalization size, resulting in the increased bandgap of the CNDs^39^. In Fig. 2h, absorption and PL emission spectra show a clear blue shift with decreasing sample size. The absorption spectra demonstrate the n–π* transition from C=N and the π–π* transition from C=C in the range of 280 to 350 nm^40,41^. The absorption peaks from the n–π* transition blueshift to 248 nm, and the π–π* transition blueshifts to 204 nm for CNDs-1@NaCNO, and the peaks further blueshift to 244 and 200 nm for CNDs-2@NaCNO.

It is important to explore the luminescence dynamic process of the CNDs in the UV region, therefore, the PL lifetime profiles of the CNDs@NaCNO synthesized at 300 and 400 °C were measured (Fig. S6). The PL lifetime profiles of CNDs-2@NaCNO indicate a lifetime of 6.4 ns, which is longer than that of CNDs-1@NaCNO (2.9 ns). A high crystallinity decreased the vibration and rotation of the bonding in the CNDs-2, which decreased the nonradiative dissipation of the carriers, thus increasing the PL QY of the CNDs. For a better understanding of the UV emission properties of CNDs-2@NaCNO, the corresponding synchronous scanning spectrum at 200–450 nm is shown in Fig. S7a. The maximum emission intensity at 342 nm corresponds to a strong UV emission caused by the 322 nm excitation. Similarly, at 312 nm with a step length of 30 nm, the highest emission intensity was observed at 342 nm (Fig. S7b). For completeness, the emission intensities variation of the corresponding synchronous scan spectra is shown in Fig. S7c; it is worth noting that the spectra show a dominant emission with a peak at approximately 342 nm. Thus, the CNDs synthesized at higher temperatures have smaller sizes, higher crystallinities and larger bandgaps, leading to decreases in vibration/rotation and exciton confinement, and thus, efficient UV emission is achieved.

**Fig. 3:**
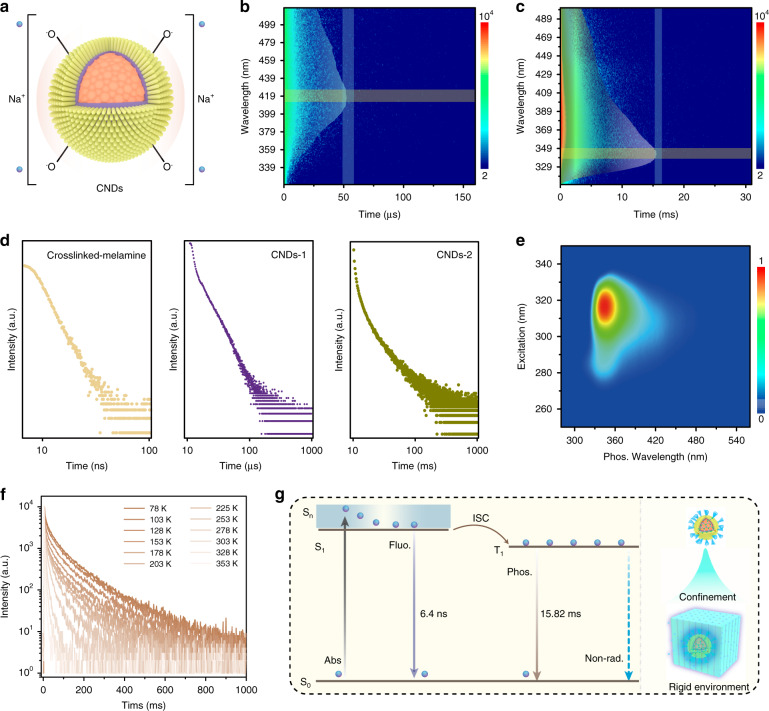
Phosphorescence characterization and a proposed UV phosphorescence mechanism. **a** Schematic diagram of CND confinement through ionic bonding. **b** Time-resolved spectra of CNDs-1@NaCNO and (**c**) CNDs-2@NaCNO. **d** Emission lifetime decay curves of crosslinked melamine@Na_2_CO_3_ (collected at 430 nm), CNDs-1@NaCNO (collected at 415 nm), and CNDs-2@NaCNO (collected at 348 nm). **e** Excitation-phosphorescence counterplots of CNDs-2@NaCNO. **f** Temperature-dependent emission decay plots of CNDs-2@NaCNO recorded at 348 nm. **g** A transition model of the UV phosphorescent CNDs

Interestingly, the CNDs confined in the NaCNO matrix show UV phosphorescence in addition to UV fluorescence. Some -NH_2_ on the CND surfaces were substituted by -OH with increasing synthesis temperature, and the hydroxyls can ionize to form multiple ionic bonds with Na^+^, as shown in Fig. 3a. To further study the phosphorescence properties, the time-resolved phosphorescence spectra of CNDs-1@NaCNO and CNDs-2@NaCNO were measured, as shown in Fig. 3b, c. The CNDs-1@NaCNO exhibited a luminescence peak at 417 nm with a lifetime of 61.1 μs under the excitation of 290 nm light (Fig. S8a), which originated from the recombination of triplet excitons. The triplet excited states of the CNDs can be efficiently stabilized through the strong confinement of the NaCNO crystal. The energy difference (Δ*E*_*ST*_) between the fluorescence and phosphorescence of CNDs-1@NaCNO is 0.45 eV (Fig. S9a). For the CNDs-2 with a small size, the phosphorescence peak blueshifted to 348 nm, and the lifetime increased to 15.82 ms (Fig. S8b). The Δ*E*_*ST*_ of CNDs-2@NaCNO decreased to 0.12 eV (Fig. S9b), which is favorable for the triplet exciton population. The higher-crystallinity carbon core and small size of CNDs-2 further decreased the vibration/rotation and enlarged the bandgap of the CNDs, leading to efficient UV phosphorescence. For clarity, the emission lifetime decay spectra of crosslinked melamine@Na_2_CO_3_, CNDs-1@NaCNO, and CNDs-2@NaCNO collected at 430, 417, and 348 nm are shown in Fig. 3d. In addition to the blueshift of the emission, the lifetimes of the CNDs can be adjusted from nanoseconds to microseconds and milliseconds. In particular, the phosphorescence spectra in Fig. 3e revealed an excitation-independent phosphorescence property of CNDs-2@NaCNO with the excitation wavelength ranging from 270 to 330 nm.

Lifetime is one of the most important parameters for phosphorescence; thus, the temperature-dependent phosphorescence lifetime decay curves at 348 nm of CNDs-2@NaCNO were recorded from 78 to 353 K to further study the influencing factor of the phosphorescence lifetime, as shown in Fig. 3f. The phosphorescence lifetimes (Fig. S10) increased gradually as the recording temperature decreased, showing a strong temperature-dependent phosphorescence property. The phosphorescence QY and lifetime are related to the rates of the corresponding radiative recombination and nonradiative recombination, which can be depicted by the following Equations^42^:

1 $$\tau _p = 1/[k_p + {{\Sigma }}k_n]$$

2 $${\it{\Phi }}_p = k_p/[k_p + {{\Sigma }}k_n]$$

where *τ*_*p*_ is the lifetime; and *k*_*p*_ and *k*_*n*_ are the radiative recombination and nonradiative recombination rates, respectively. The *k*_*n*_ of CNDs-2@NaCNO is 52.9 s^−1^ at room temperature and 4.88 s^−1^ at 78 K, as shown in Table S2. The increase in the vibration/rotation of the CNDs is the main reason for the increased *k*_*n*_ at elevated temperatures^43,44^. Thus, the spatial confinement-induced decrease in *k*_*n*_ plays a key role in the UV phosphorescence of the CNDs, and UV phosphorescent CNDs with longer lifetimes can be achieved in a more rigid environment. A transition model of the UV phosphorescent CNDs is shown in Fig. 3g by combing steady and transient spectra. The electrons in the ground state (*S*_0_) of the CNDs will be excited to the singlet excited state (*S*_1_) under the excitation of 310 nm, and they have a closer distance in space due to spatially symmetric electron wave functions. Some electrons transition to triplet states through the ISC process, and the electrons thus separate according to the Pauli Exclusion Principle^45^. The electrons that are closer together have larger repulsive energy; thus, the lowest singlet states (*S*_1_) have higher energies compared with the lowest triplet states (*T*_1_)^46,47^. The electrons in *S*_1_ and *T*_1_ return to the ground state (*S*_0_) within 6.4 ns and 15.8 ms, releasing UV photons. In addition, the photo, temporal, and ambient stabilities of CNDs-2@NaCNO were evaluated, as shown in Fig. S11. There was no significant loss of the phosphorescence intensity under continuous excitation, and an oxygen atmosphere was observed when the samples were stored in ambient conditions for 30 days, suggesting their relatively high stability.

**Fig. 4:**
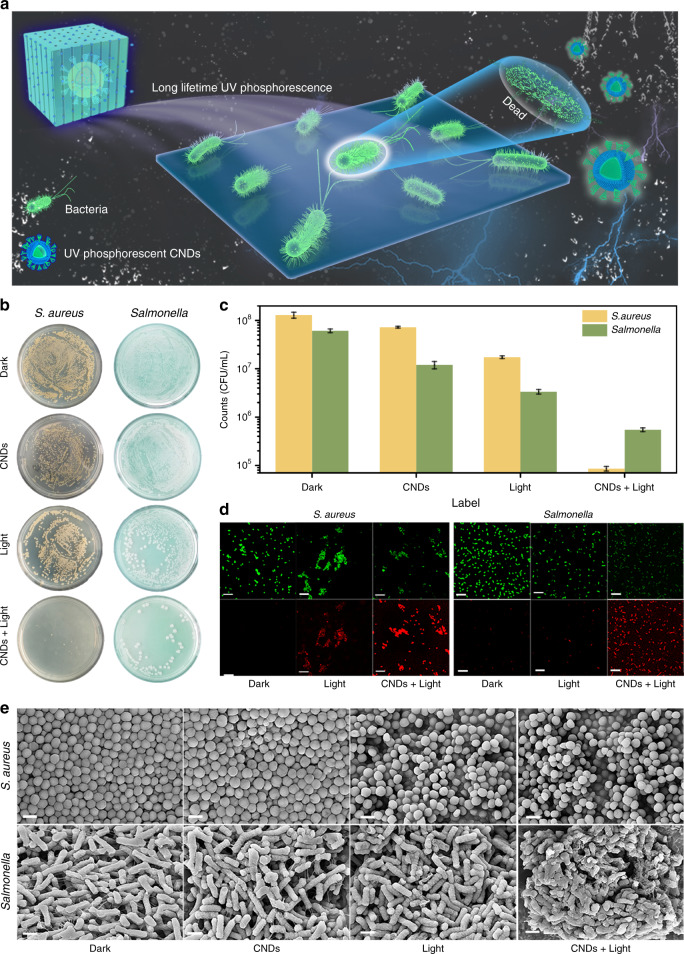
Demonstration of the UV phosphorescent CNDs for antibacterial applications. **a** Schematic diagram of the interaction between UV phosphorescence and bacteria. **b** Images of the flat colony counting results under different conditions. **c** Number of bacterial counts (CFU mL^−1^) in panel (**b**). **d** CLSM images of *S. aureus* and *Salmonella* after treatment with CNDs-2@NaCNO under excitation of pulsed light for five cycles (one cycle: irradiation six times within 1 min, excitation light: 310 nm, 7 mW cm^−2,^ 0.1 Hz); scale bar is 20 µm. **e** SEM images of the *S. aureus* and *Salmonella* under different conditions; scale bar is 1 μm

From the above results, one can see that CNDs-2@NaCNO can emit UV phosphorescence and has potential antibacterial applications. Upon irradiation with pulsed light, CNDs-2@NaCNO was activated and emitted UV photons for a long time. Thus, the interaction between the CNDs-2@NaCNO and bacteria was strengthened, leading to the death of the bacteria. A schematic diagram is illustrated in Fig. 4a. The biocompatibility of the CNDs-2@NaCNO was evaluated through histological analysis before the antibacterial test, and no obvious tissue lesions were observed (Fig. S12), indicating good biocompatibility. Moreover, the cytotoxicity of CNDs-2@NaCNO was assessed through MTT cell proliferation-toxicity assays, as shown in Fig. S13.

For a wide dose range of CNDs-2@NaCNO (0–200 ug/ml), the cell viability percentages of the human umbilical vein endothelial (HUVEC) cells and human hepatocellular carcinoma (HepG2) cells were all above 90% of their original values after 24 h of incubation, indicating that CNDs-2@NaCNO has negligible cytotoxicity on normal cells. *S. aureus* and *Salmonella* were chosen to evaluate the antibacterial performance of CNDs-2@NaCNO. The long-lifetime UV photon cytotoxicity effects of CNDs-2@NaCNO under different conditions were determined by the analysis of *S. aureus* and *Salmonella* bacterial colony images, which are shown in Fig. 4b. Both *S. aureus* and *Salmonella* grew and multiplied freely on agar plate no matter under both dark and light pulse irradiation conditions. In contrast, hardly any bacterial colonies of *S. aureus* and *Salmonella* persisted under treatment with CNDs-2@NaCNO and pulsed light (0.1 Hz) with 7 mW cm^−2^. The bacteria treated with CNDs-2@NaCNO under dark conditions continued to increase, indicating that bacteria inactivation was caused by the emitted high-energy photons. For quantitative characterization, bacterial counts of *S. aureus* and *Salmonella* under different conditions are shown in Fig. 4c and Fig. S14. The death of the bacteria increased rapidly with an increasing number of irradiation cycles (Fig. S15a), indicating that the longer interaction between the emitted UV photons and bacteria can cause the death of more bacteria. The sterilization efficiency can reach more than 90% within three cycles and it is close to 100% after five cycles, as shown in Fig. S14b, c. The above results indicate that in terms of long-lifetime UV photon production, the number of UV photons of CNDs-2@NaCNO gradually increased due to the number of triggers by increasing irradiation and CNDs-2@NaCNO can be used as an antibacterial nanomaterial. Furthermore, a relative antibacterial test with NaCNO as a control group was performed for *S. aureus* (Fig. S16), and the cytotoxicity effect of NaCNO on *S. aureus* was negligible. In addition, confocal laser scanning microscope (CLSM) images of the bacteria stained with propidium iodide (PI) and SYTO 9 fluorescent indicator are shown in Fig. 4d. The bacteria stained by the PI indicator with damaged membranes showed red emission, while bacteria with intact membranes showed green emission due to SYTO 9 staining. For the bacteria incubated with CNDs-2@NaCNO under excitation with pulsed light, most of them showed bright red fluorescence in comparison to other groups, demonstrating the high antibacterial ability of CNDs-2@NaCNO. Furthermore, the scanning electron microscopy images in Fig. 4e demonstrates the morphological changes of *S. aureus* and *Salmonella*. The bacteria treated with CNDs-2@NaCNO under excitation with pulsed light showed obvious membrane damage, while the bacteria in the control group remained intact structure. These results indicated that the inactivation of bacteria is due to the rupture of bacterial membranes caused by exposure to high-energy photons.

## Discussion

In conclusion, we proposed design principles for UV phosphorescent CNDs: (1) The formed CNDs should have a small conjugation size, and first-principles density functional theory calculations can provide qualitative guidance for precursor selection; (2) Heteroatoms with lone pairs of electrons should be included in the CNDs to provide enough ISC driving force; and (3) An in-situ generated confinement environment is favorable considering the instability of triplet excitons. Of course, deeper physical effects should be further investigated. Under the guidance of the proposed design principles, UV phosphorescent CNDs with peaks centered at 348 nm and lifetimes of 15.8 ms have been realized. The phosphorescence wavelength can be tuned from 430 to 348 nm by decreasing the size of the CNDs and suppressing the vibration/rotation of the CNDs with the confinement of the triplet excitons by the NaCNO crystal. The highest UV phosphorescence QY of the CNDs reached 16.2%. As a demonstration, the UV phosphorescent CNDs were used to inactivate gram-negative/positive bacteria, and the antibacterial efficiency reached up to 99.9% against *S. aureus* and *Salmonella*. These results provide a rational design strategy for UV phosphorescent CNDs, and demonstrate promise for the potential antibacterial applications of CNDs.

## Materials and methods

### Chemicals and materials

Melamine and sodium hydroxide were purchased from Shanghai Macklin Chemistry Co. Ltd. and used without further purification.

### Synthesis of crosslinked melamine@Na_2_CO_3_, CNDs-1@NaCNO, and CNDs-2@NaCNO

First, 0.5 g melamine and 0.1 g NaOH precursors were mixed in a mortar and ground uniformly for 10 min, and then the resulting solid mixtures of melamine and NaOH were calcined at 200 °C in a muffle furnace for 10 h with a heating rate of 20 °C min^−1^. The generated products were denoted as crosslinked melamine@Na_2_CO_3_. For CNDs-1@NaCNO, the ratio between o-melamine and NaOH was 1:1 (0.5 g melamine and 0.5 g NaOH), and CNDs-1@NaCNO powders was obtained after heating at 300 °C for 10 h_._ For CNDs-2@NaCNO, the reaction temperature was further increased to 400 °C, and after 10 h, CNDs-2@NaCNO powders were obtained. The obtained sample powders were used for further experimentation.

### First-principles density functional theory calculations

The optimization of the ground state geometries of the UV phosphorescent CNDs was performed through density functional theory (DFT), and identity approximation was carried out using the B3LYP functional together with the chain of spheres exchange method. Due to the different noncovalent interactions, Grimme’s dispersion correction was introduced in the calculation. Time-dependent density functional theory and Grimme’s dispersion (D3) correction were used to calculate the excitation energies. Further confirmation of the nature of the structure with minimum energy (resting point) was performed by conducting frequency analysis. DFT calculations were performed using the Visual Molecular Dynamics package and Gaussian 09 package.

### Animal experiments

BALB/c mice (6–8 weeks, female) were purchased from Hunan Shrek Jingda experimental animal Co., Ltd. All the experiments were in compliance with the policies of the National Ministry of Health. All animal procedures were performed following a protocol approved by the Institute of Drug Discovery & Development of Zhengzhou University (syxk (yu) 2018-0004).

### Histological experiments and cytotoxicity measurements

CNDs-2-NaCNO (1000 μg/ml) were injected intravenously into mice for 1 day and 7 days and the control group of mice was injected with normal saline. On the 7th day, the organs (heart, kidney, bladder, liver, and lung,) from all wound sites were excised for histopathological evaluation.

The cytotoxicity was measured via MTT cell proliferation-toxicity assays. Human umbilical vein endothelial (HUVEC) cells and human hepatocellular carcinoma (HepG2) cells were seeded onto 96-well plates at 37 °C. Twenty-four hours later, CNDs-2-NaCNO solutions of different concentrations (0–200 μg/mL^−1^) were added to each well. Subsequently, the MTT solution (10 µL) was added to each well of the plate after further incubation for 24 h. Then, the optical density properties at 492 nm of each well were recorded to evaluate the cell viability.

### Antibacterial experiments

The antibacterial properties were evaluated by using *S. aureus* and *Salmonella as* gram-positive/negative bacteria. A single colony of *S. aureus* was added to a 20 ml Luria-Bertani (LB) medium plate by using an inoculating loop. Then, the bacterial suspensions in these plates were incubated to achieve a bacterial solution (~10^9^ CFU/mL) with exponential growth at 37 °C for 13 h. The obtained bacteria were diluted to 10^8^ CFU/mL, and the diluted bacterial suspension (100 μL) was evenly dispersed on LB agar plates in which CNDs with different masses (0, 0.001, 0.002, 0.004, 0.006, 0.008, and 0.010 g) had been distributed. Subsequently, these plates were irradiated by pulsed 310 nm light for five cycles (one cycle: irradiation six times within 1 min, excitation light: 310 nm, 7 mW cm^−2^, each interval is 10 s). After that, they were transferred to an incubator. Similarly for *Salmonella*, a single colony was taken in 20 ml of LB medium at 180 rpm for 13 h to reach a bacterial concentration of 10^8^ CFU/ml. Then the same procedure as above was carried out. Finally, the colony counting method, SEM observation method, and fluorescence staining method were used to analyze the antibacterial properties of the CNDs.

### Structural and optical characterization

The microstructures of all the CNDs were characterized by transmission electron microscopy (TEM, JSM-6700), and X-ray diffraction (XRD) patterns were collected with a Bruker D8 Discover (Germany) X-ray diffractometer. For the measurement of the fluorescence and phosphorescence of the samples, the spectra were characterized using a Hitachi F-7000 PC spectrophotometer. The lifetimes were measured using an FLS-1000 spectrometer equipped with a microsecond flash lamp. The absolute PL QYs were measured using an absolute photoluminescence quantum yield FLS-1000 equipped with an integrating sphere under ambient conditions. UV–vis absorption spectra were performed using a Hitachi UH4150 UV–vis spectrophotometer. The FTIR spectra of the samples were recorded using a Bruker VERTEX-70 FTIR spectrometer. The fluorescence imaging of bacteria was performed by using a Leica TCS SP8 STED 3X laser scanning confocal microscope.

## Supplementary information

Supplementary information
