# Supplementary material for: Ultraviolet phosphorescent carbon nanodots
Source: Light Sci Appl. 2022 May 20;11:146. doi: 10.1038/s41377-022-00837-1 (PMC9122994; doi:10.1038/s41377-022-00837-1)
Supplement: Supplementary file 1 — Supplementary information [file 41377_2022_837_MOESM1_ESM.doc]

Supplementary Information for

**Ultraviolet Phosphorescent Carbon Nanodots**

Shiyu Song, Kaikai Liu*, Qing Cao, Xin Mao, Wenbo Zhao, Yong Wang, Yachuan Liang, Jinhao Zang, Qing Lou, Lin Dong and Chongxin Shan*

Henan Key Laboratory of Diamond Optoelectronic Material and Devices, Key Laboratory of Material Physics, Ministry of Education, School of Physics and Microelectronics, Zhengzhou University, Zhengzhou 450001, China.

*Email: liukaikai@zzu.edu.cn; cxshan@zzu.edu.cn.

**Table of Content**

1. **Figure S1.** Computational optical properties of the configurations of CNDs.
2. **Figure S2.** TEM images of CNDs.
3. **Figure S3.** XRD patterns of CNDs.
4. **Table S1.** XPS element content of CNDs
5. **Figure S4.** FT-IR spectra of CNDs.
6. **Figure S5.** XPS spectra of CNDs.
7. **Figure S6.** Fluorescence lifetime decay spectra of CNDs.
8. **Figure S7.** Synchronous scan spectra of CNDs.
9. **Figure S8.** Phosphorescence lifetime fitting spectra of CNDs.
10. **Figure S9.** Photoluminescence and phosphorescence spectra of CNDs at 77 K.
11. **Figure S10.** Phosphorescence decay lifetimes of CNDs from 78 K to 353K.
12. **Table S2.** Dynamic photo-physical parameters of the CNDs.
13. **Figure S11.** Phosphorescence Stability of CNDs.
14. **Figure S12.** Histological analysis of CNDs.
15. **Figure S13.** Cell viability of CNDs.
16. **Figure S14.** Antibacterial efficiency against *S. aureus* and *Salmonella* of CNDs.
17. **Figure S15.** Antibacterial properties of CNDs.
18. **Figure S16.** Antibacterial efficiency of NaCNO.


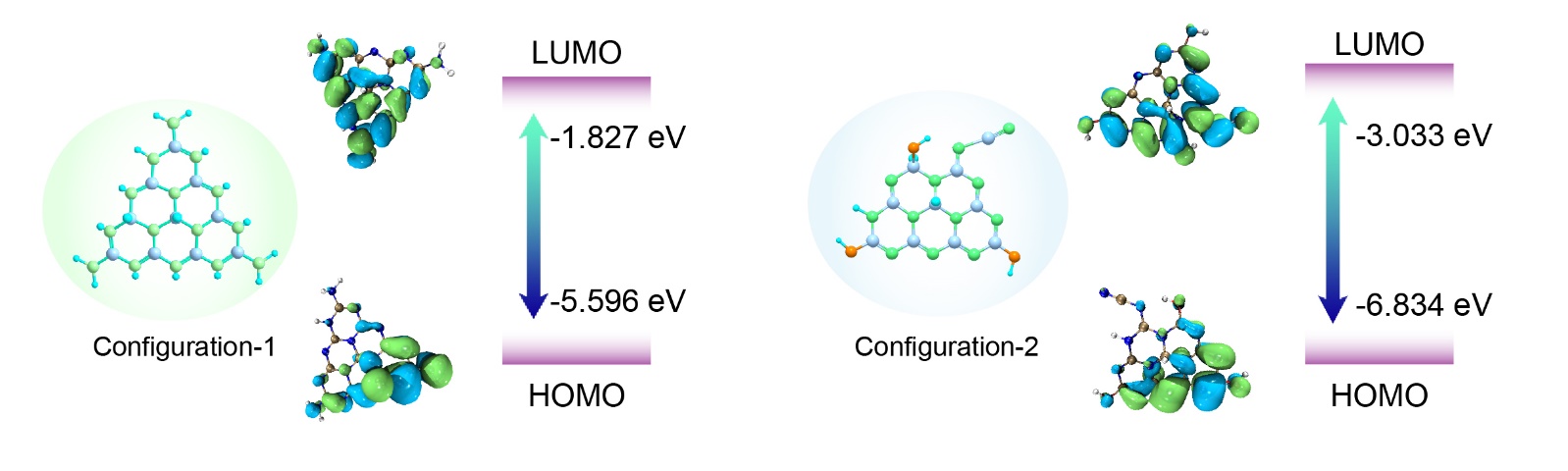
Figure S1. The HOMO and LUMO of two configurations of the CNDs.


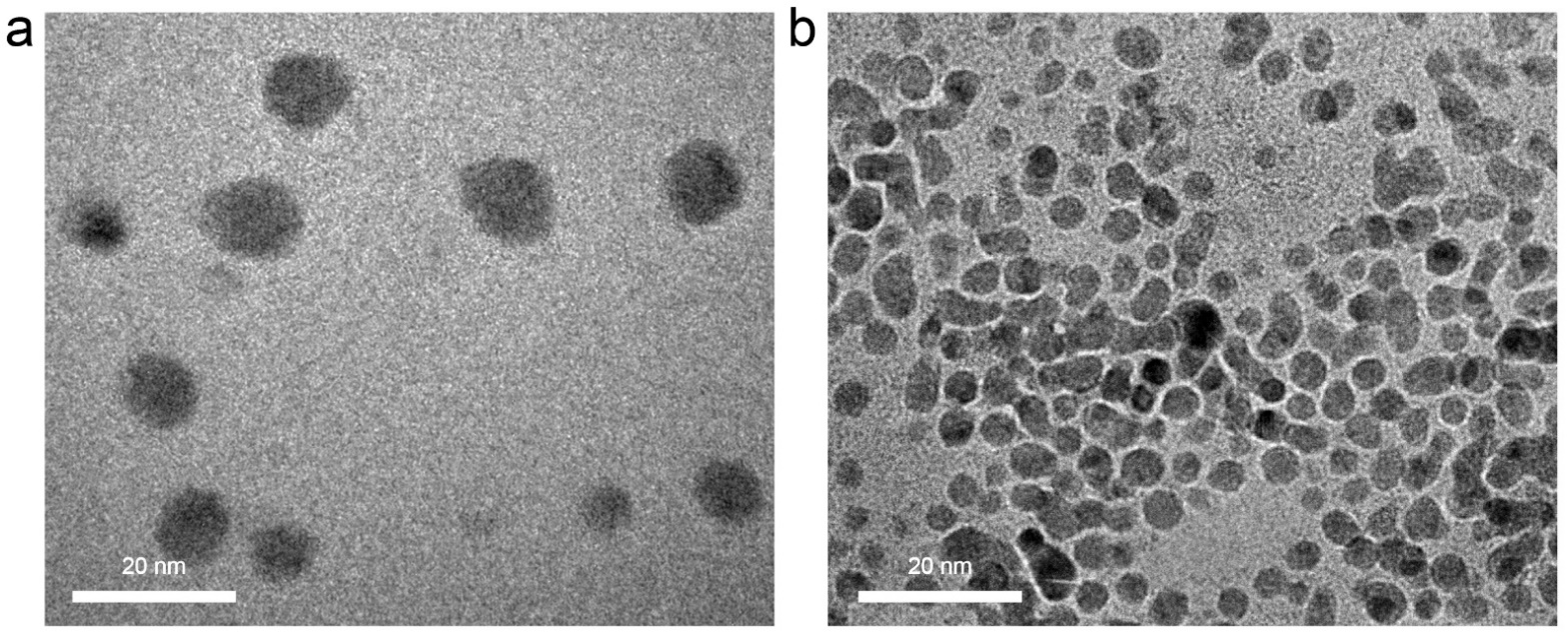


Figure S2. TEM images of the (a) formed CNDs at 300 ^o^C and the (b)formed CNDs at 400 ^o^C.


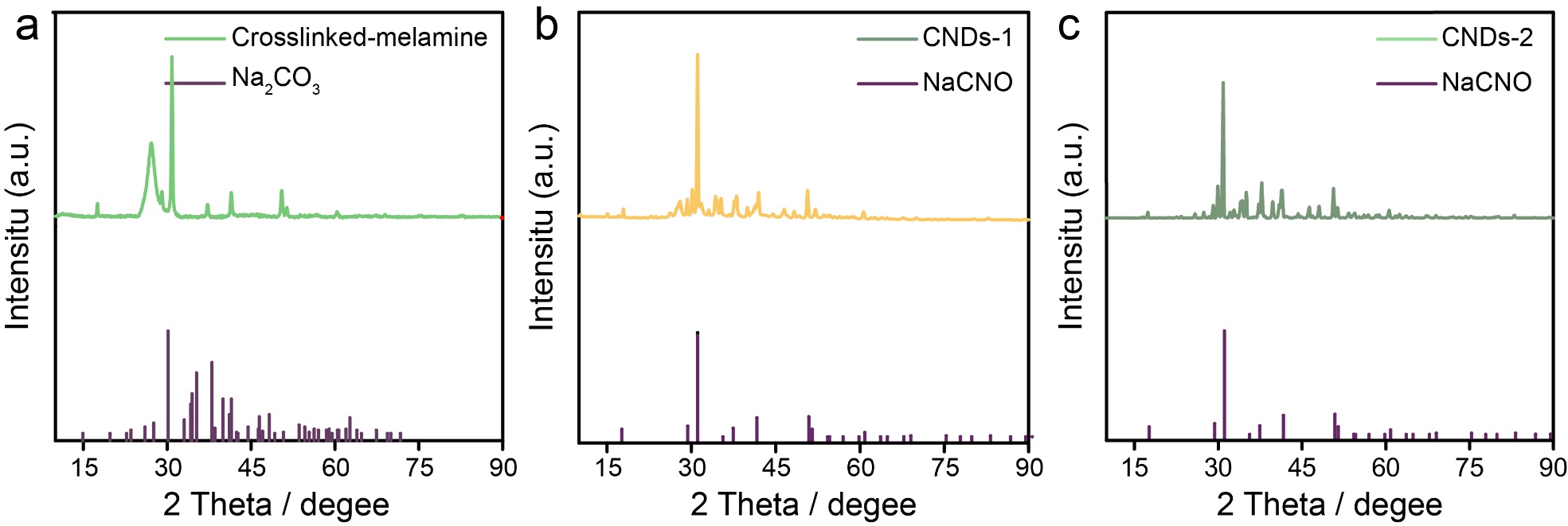


Figure S3. X-ray diffraction (XRD) patterns of the (a) crosslinked-melamine@Na_2_CO_3_, (b) CNDs-1@NaCNO, and (c) CNDs-2@NaCNO.

Table S1. The element content of the crosslinked-melamine@Na_2_CO_3_, CNDs-1@NaCNO and CNDs-2@NaCNO.


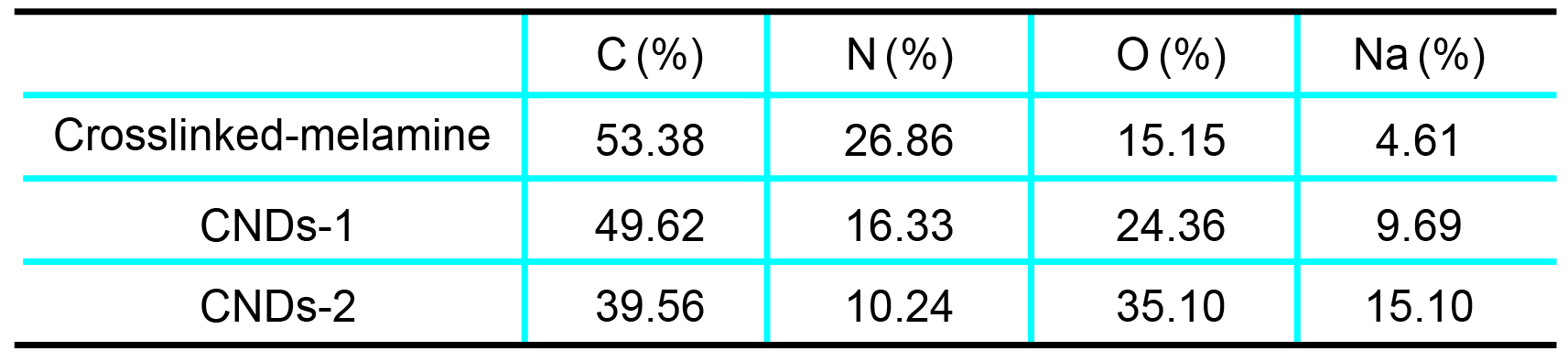


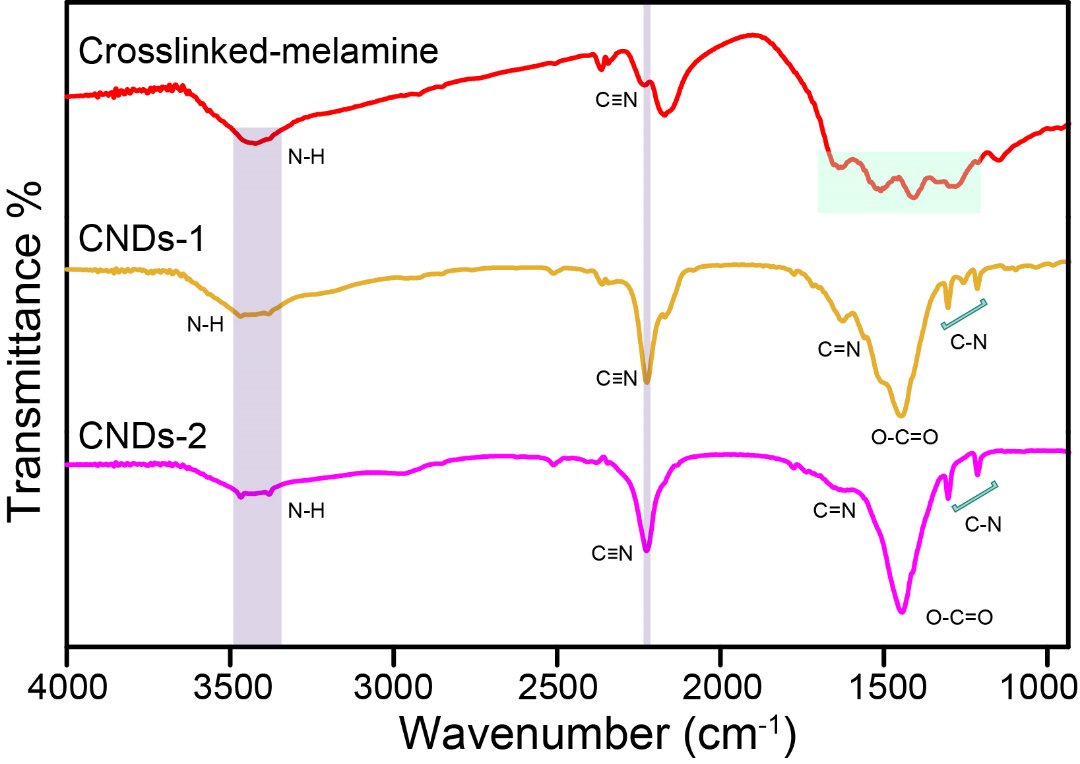


Figure S4. FT-IR spectra of the crosslinked-melamine@Na_2_CO_3_ (red line), CNDs-1@NaCNO (green line) and CNDs-2@NaCNO (blue line).


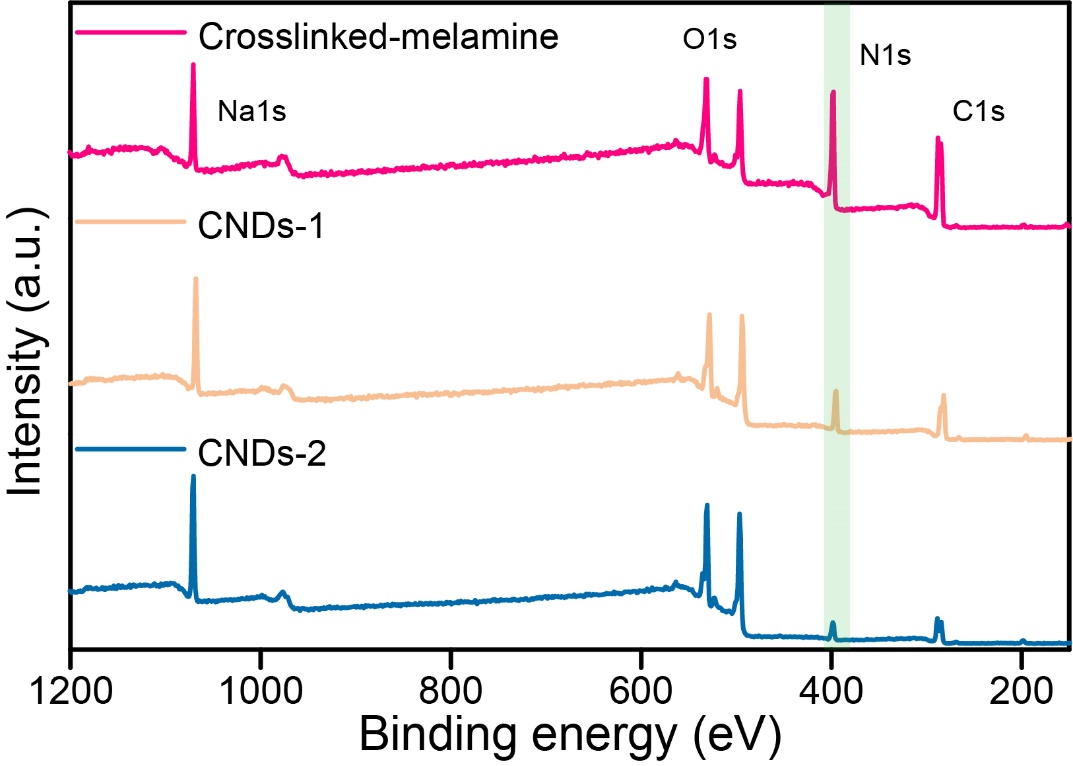


Figure S5. XPS spectra of the crosslinked-melamine@Na_2_CO_3_, CNDs-1@NaCNO and CNDs-2@NaCNO.

.
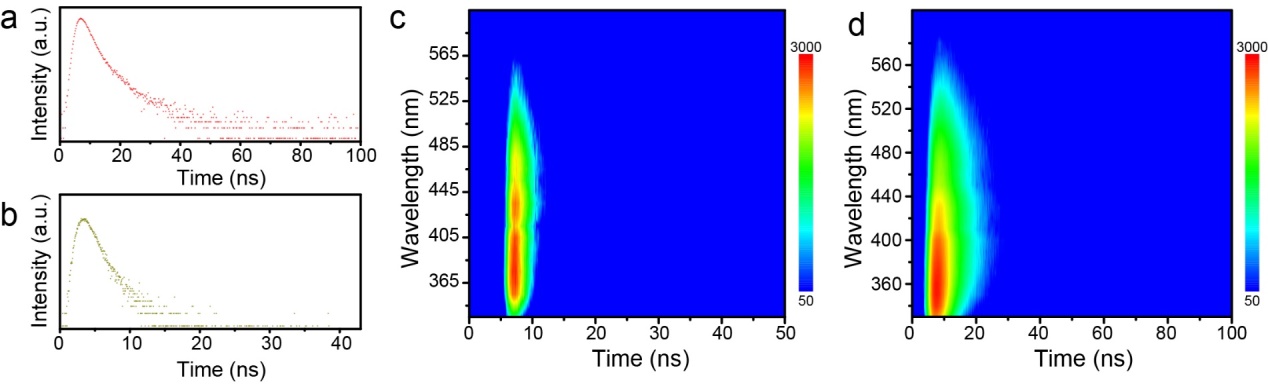


Figure S6. Fluorescence lifetime resolved luminescence decay curves of the CNDs-1@NaCNO (a) and CNDs-2@NaCNO (b). Time resolved luminescence contours of the CNDs-1@NaCNO (c) and CNDs-2@NaCNO (d).


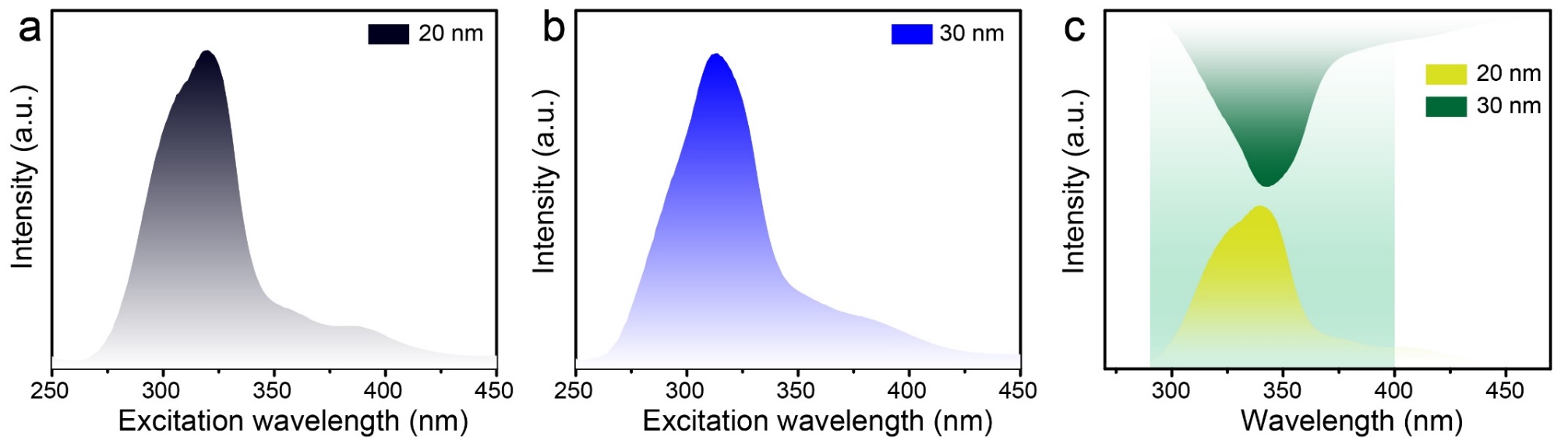


Figure S7. The synchronous scan spectra of CNDs-2@NaCNO (excitation wavelength scanning distance: 20 nm (a) and 30 nm (b)). (c) The plots of emission variation intensity of the corresponding synchronous scan spectra (a) and (b)


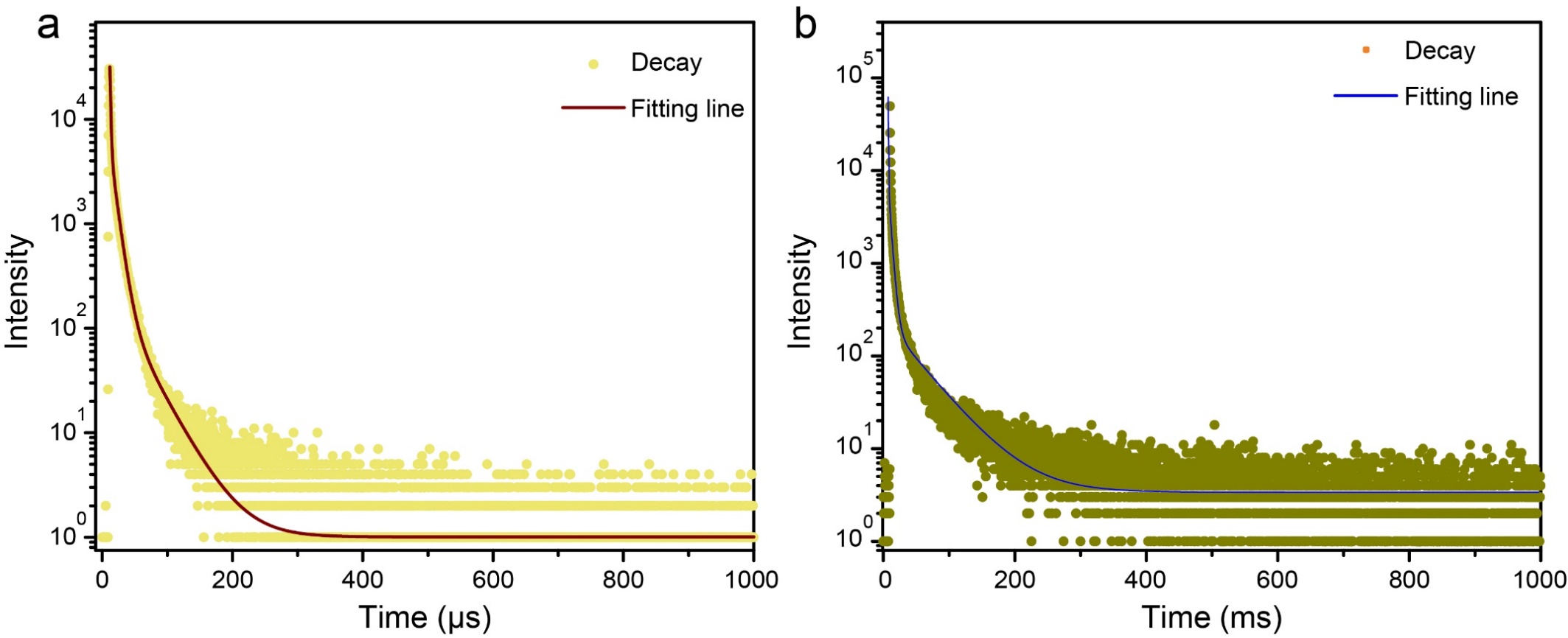


Figure S8. The time-resolved decay plots of the CNDs-1@NaCNO (a) and CNDs-2@NaCNO (b) at room temperature.


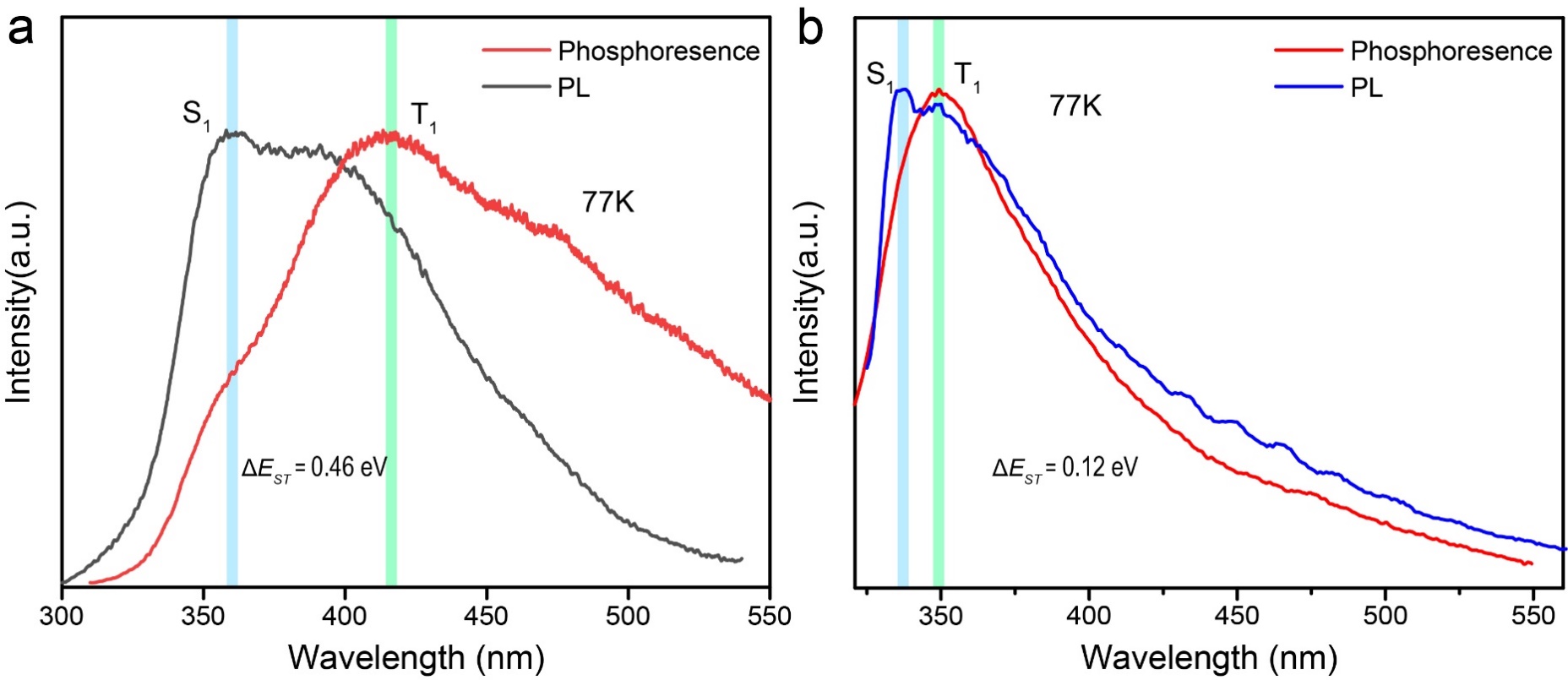


Figure S9. (a) Photoluminescence spectrum (black line) and phosphorescence spectrum (red line) of CNDs-1@NaCNO excited under 310 nm at 77 K. (b) Photoluminescence spectrum (blue line) and phosphorescence spectrum (red line) of CNDs-2@NaCNO excited under 310 nm at 77 K.


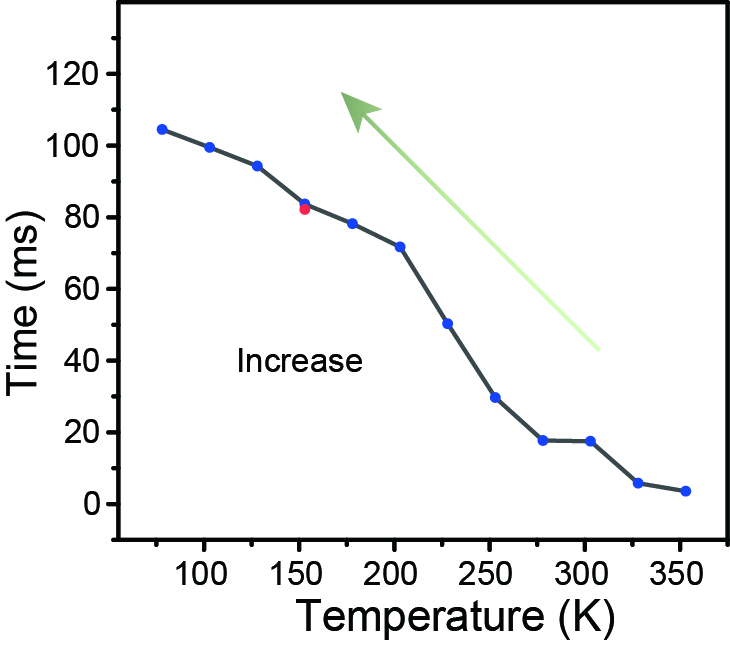


Figure S10. Plot of phosphorescence emission decay lifetimes versus temperature from 78 K to 353K

Table S2. Dynamic photo-physical parameters of the CNDs-2@NaCNO


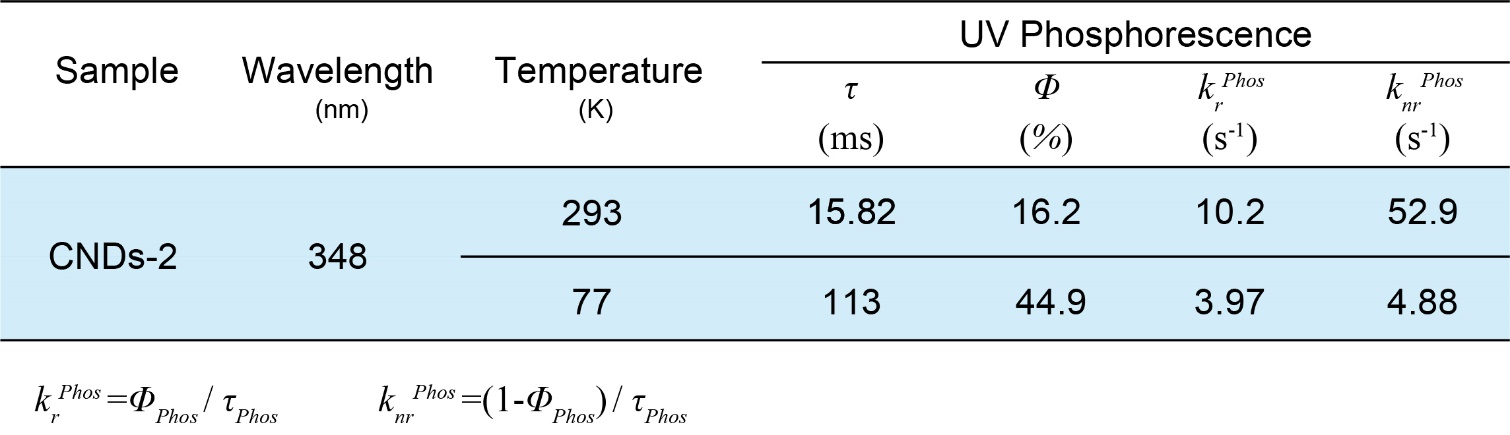


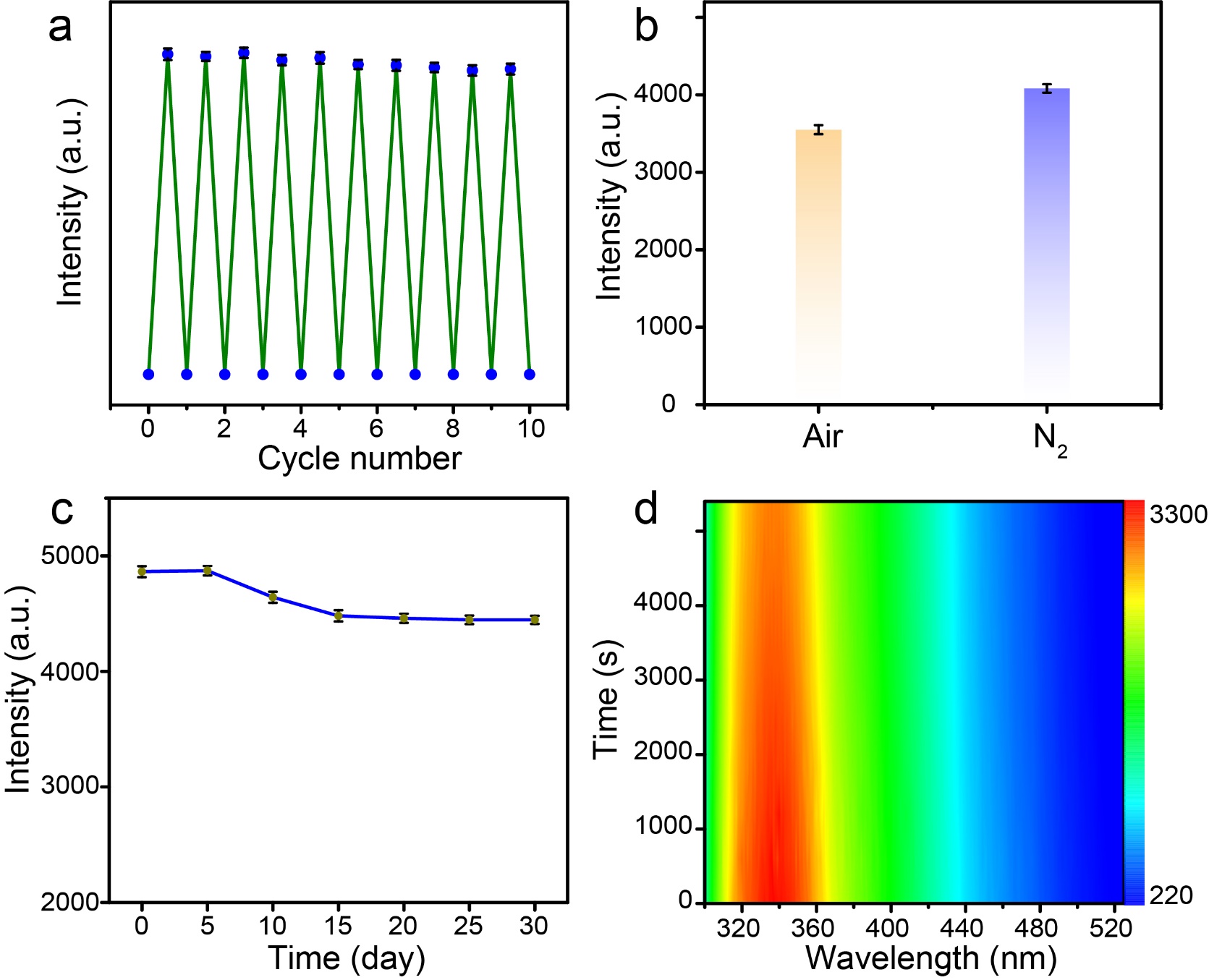


Figure S11. (a) Phosphorescence intensity of the CNDs-2@NaCNO as a function of the cycle number of illuminations. (b) The phosphorescence intensity of the CNDs-2@NaCNO purged with air or nitrogen. (c) The UV phosphorescence intensity of the CNDs-2@NaCNO versus time stored at ambient condition. (d) The UV phosphorescence photo-stability of the CNDs-2@NaCNO.


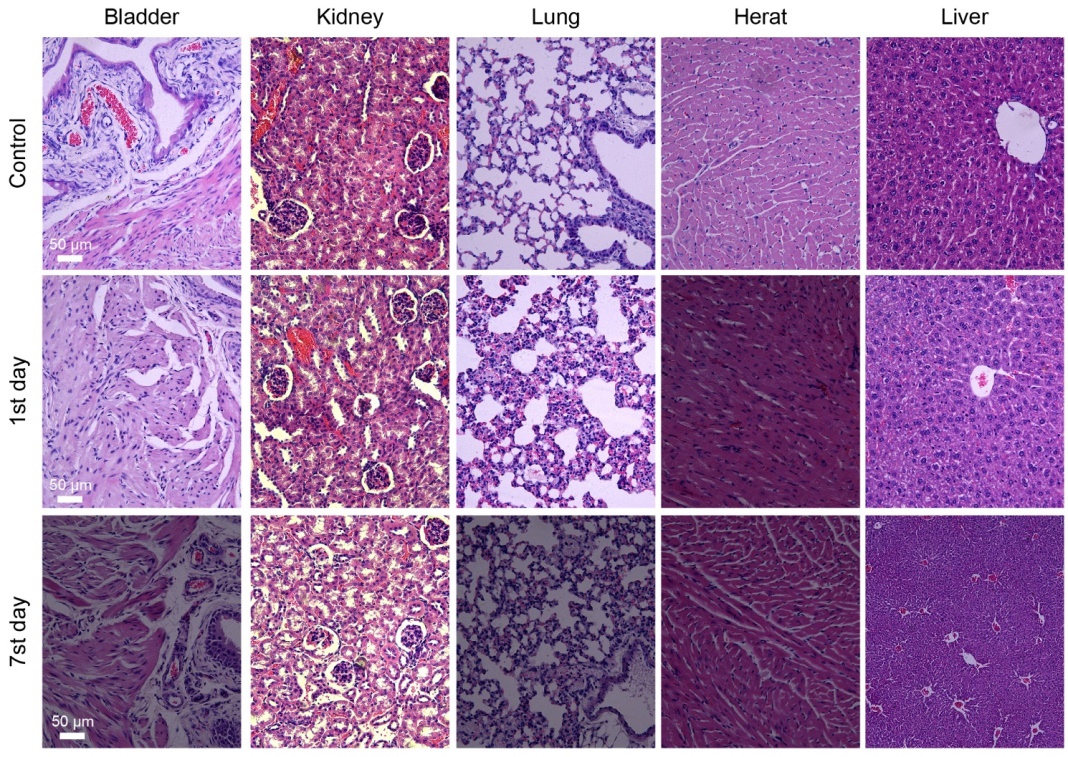


Figure S12. Hematoxylin and eosin-stained slices of bladder, kidney, lung, liver and heart tissues of the CNDs-2@NaCNO and control group (normal saline).


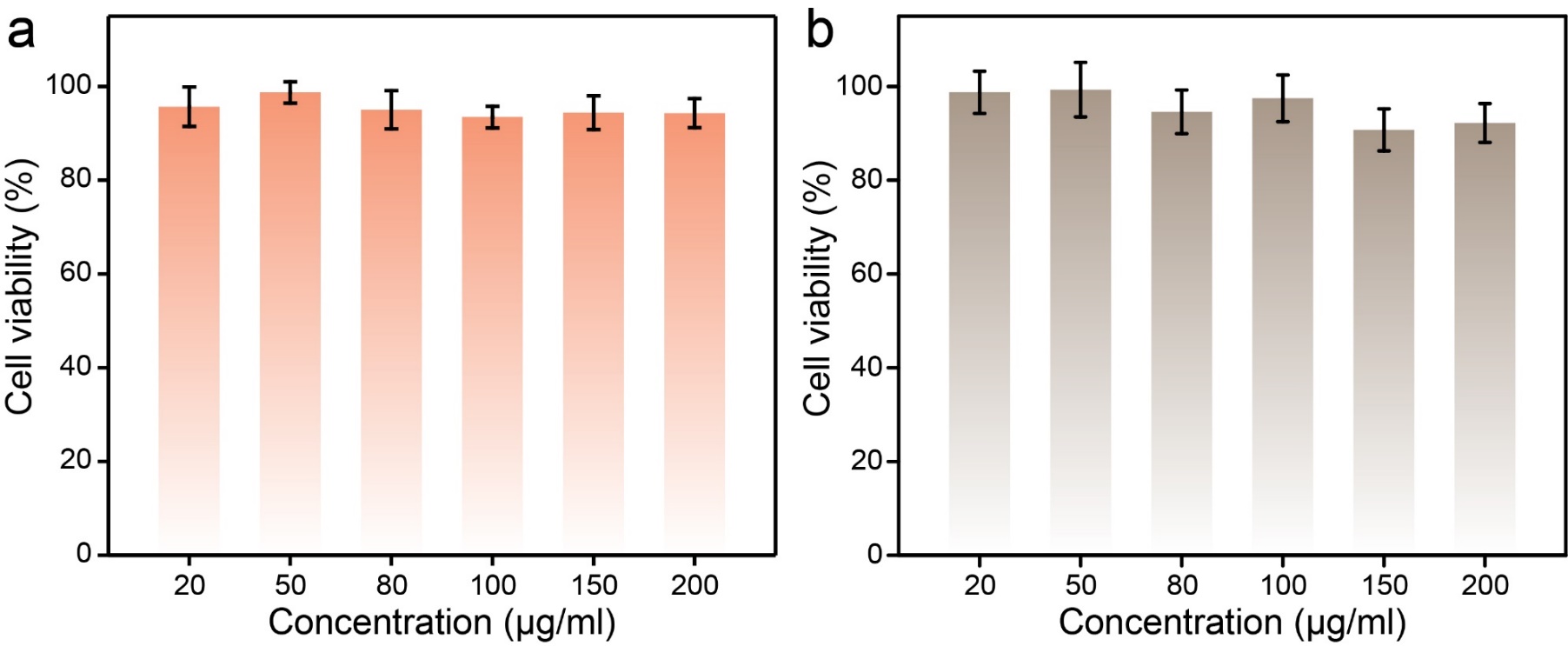


Figure S13. Cell viability of the HUVEC cells (a) and HepG2 cells (b) after incubation with CNDs-2@NaCNO of different concentrations.


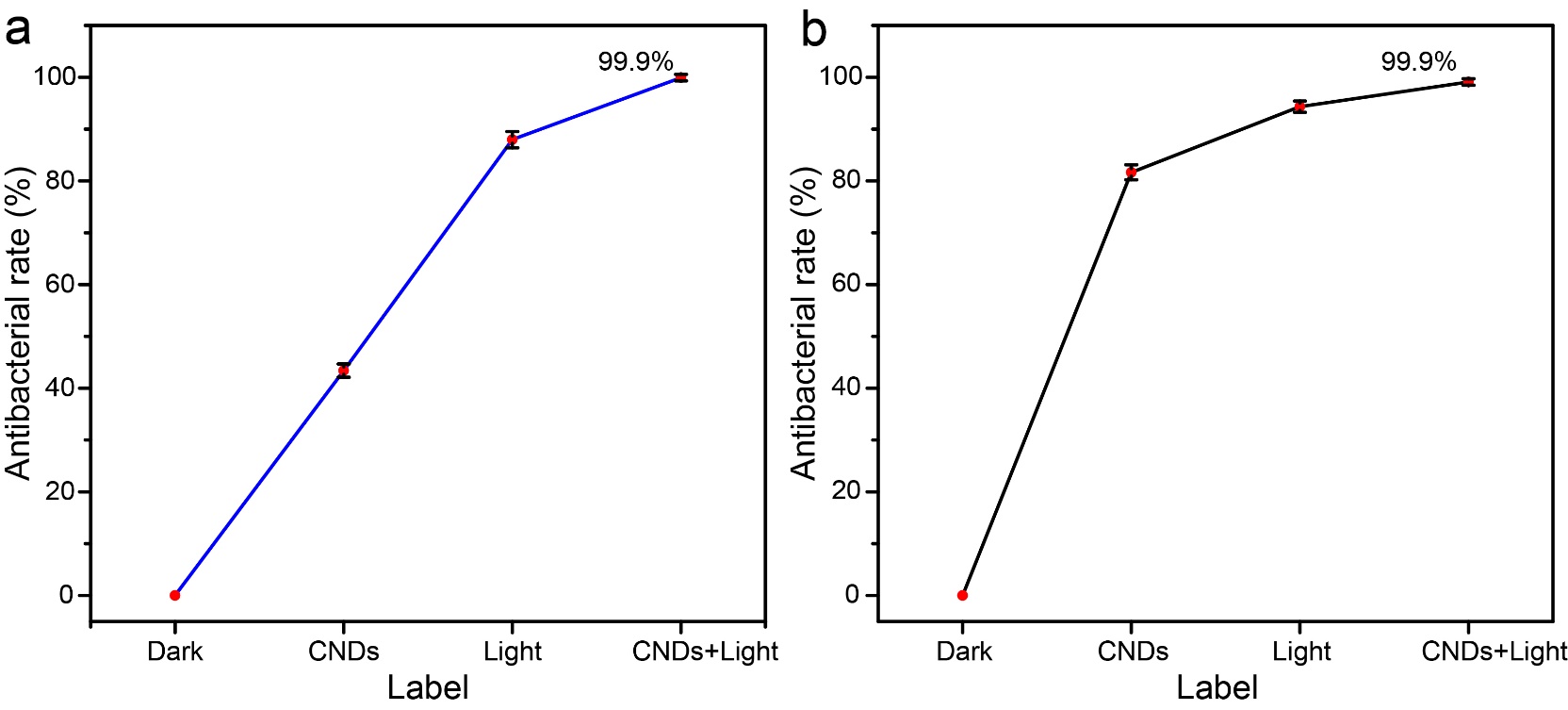


Figure S14. (a) *S. aureus* and (b) *Salmonella* inactivation rates of different groups.


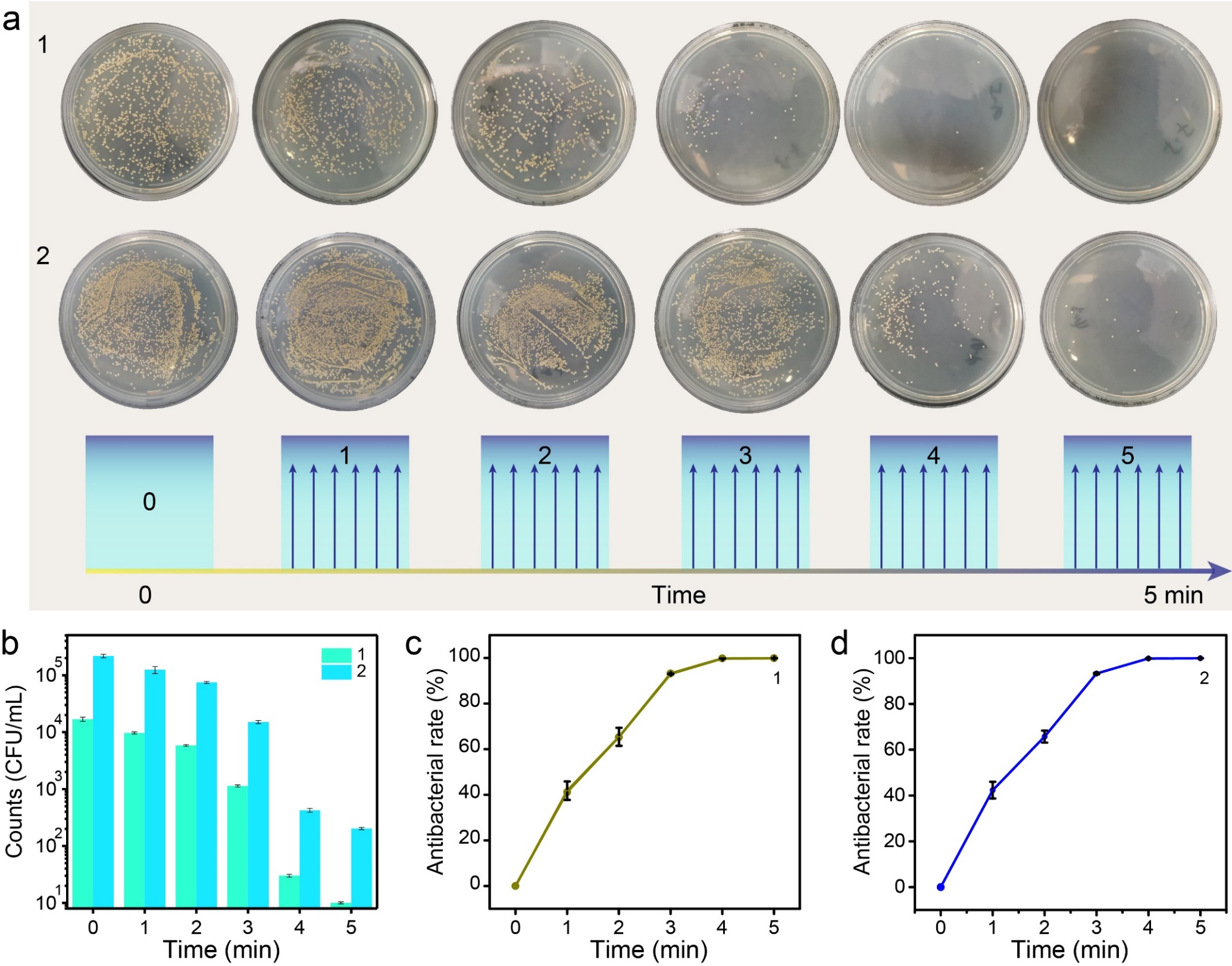


Figure S15. (a) Images of the flat colony counting results. (b) Number of the bacterial counts (CFU mL^−1^) in panel (a). (c, d) *S. aureus* inactivation rates of different groups within 5 mins.


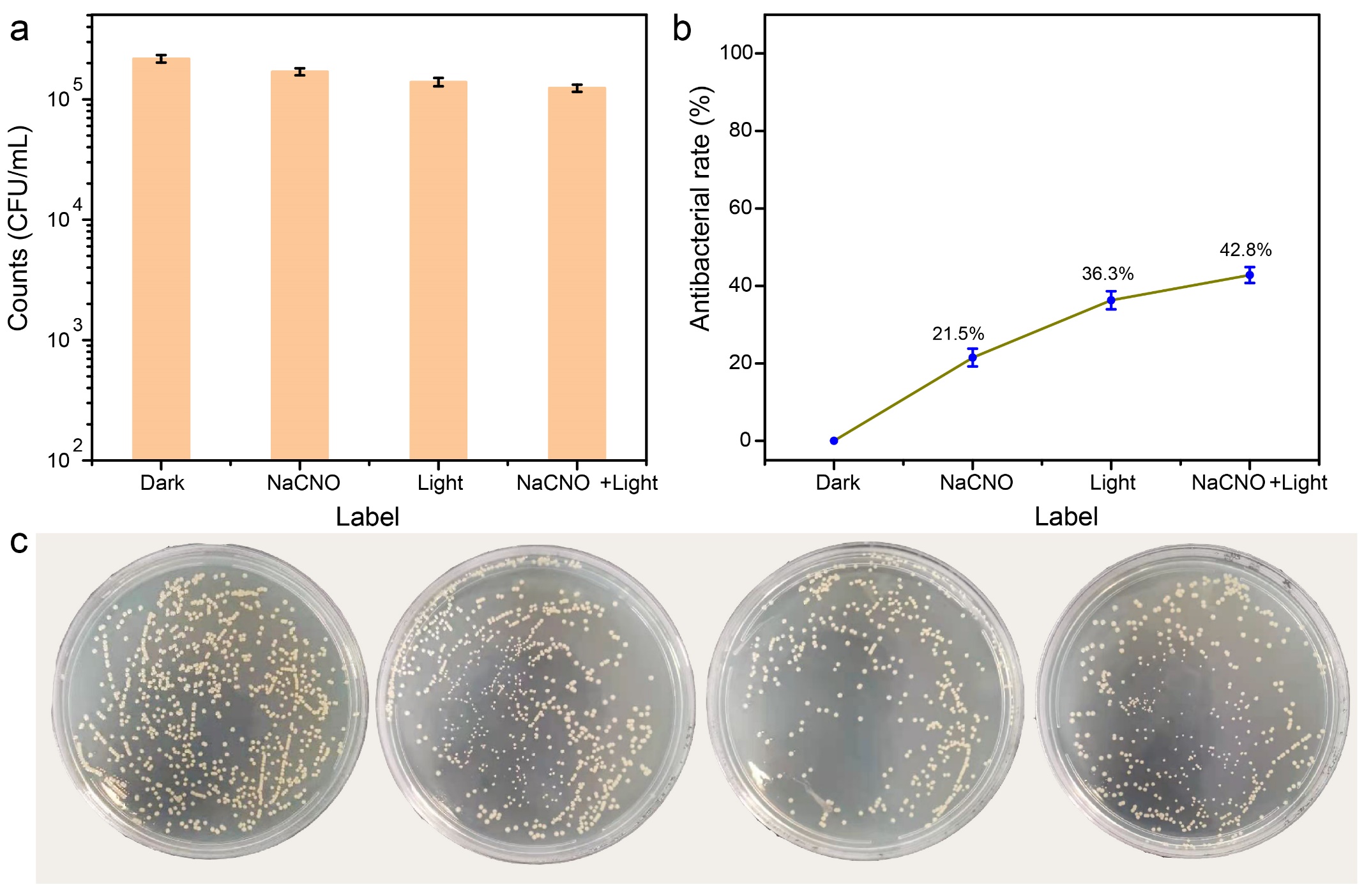


Figure S16. (a) Number of the bacterial counts (CFU mL^−1^). (b) *S. aureus* inactivation rates of the control group. (c) Images of the flat colony counting results.
